# Decrease in loop diuretic treatment from 2005 to 2014 in Swedish real-life patients with chronic heart failure

**DOI:** 10.1007/s00228-018-2574-6

**Published:** 2018-10-15

**Authors:** Pär Parén, Annika Rosengren, Tatiana Zverkova Sandström, Maria Schaufelberger

**Affiliations:** 1 Department of Internal Medicine, Sahlgrenska University Hospital/Mölndal, S-431 80 Mölndal, Sweden; 2 Department of Molecular & Clinical Medicine, Institute of Medicine, Sahlgrenska Academy/University of Gothenburg, Gothenburg, Sweden; 3 Department of Internal Medicine, Sahlgrenska University Hospital/Östra, Gothenburg, Sweden

**Keywords:** Heart failure, Outpatients, Pharmaco-epidemiology

## Abstract

**Purpose:**

Loop diuretics are recommended to treat congestive symptoms in patients with heart failure. However, observational studies have indicated that loop diuretic treatment in heart failure is associated with increased mortality. Therefore, loop diuretic discontinuation or dose reduction, when clinically possible, is recommended. Our aim was to study nationwide temporal trends in loop diuretic treatment from 2005 to 2014 in real-life patients with chronic heart failure.

**Methods:**

Data from the nationwide Swedish National Patient, Prescribed Drug and Cause of Death Registers were linked. The annual proportions of patients with chronic heart failure treated with loop diuretics from 2005 to 2014 were calculated. In addition, the annual median loop diuretic doses (DDD) in patients with chronic heart failure treated with loop diuretics from 2005 to 2014 were calculated.

**Results:**

The proportion of real-life patients with chronic heart failure treated with loop diuretics decreased from 73.2% in 2005 to 65.7% in 2014 (*p* for trend < 0.001). The median loop diuretic DDD in real-life patients with chronic heart failure decreased from 2.13 (IQR 1.09–2.77) in 2005 to 1.63 (IQR 1.09–2.25) in 2014 (*p* = 0.001 for trend).

**Conclusions:**

Loop diuretic treatment decreased from 2005 to 2014 in real-life patients with chronic heart failure. The prognostic impact of changes in loop diuretic treatment in patients with heart failure remains unclear.

**Electronic supplementary material:**

The online version of this article (10.1007/s00228-018-2574-6) contains supplementary material, which is available to authorized users.

## Introduction

Major evidence-based advances in the pharmacologic treatment of chronic heart failure (HF) have been made during the last decades. Nevertheless, not all drugs currently recommended in HF have proven prognostic benefits. Loop diuretics are frequently used to treat congestive symptoms in patients with HF, albeit the prognostic impact of this strategy is not clear due to a lack of randomized clinical trials. In fact, observational reports have suggested an association between loop diuretic treatment and increased mortality in patients with HF irrespective of ejection fraction (EF) and symptomatic severity [1–5]. Consequently, the European Society of Cardiology (ESC) guidelines on the treatment of HF have recommended loop diuretics for symptomatic relief but also reduction of the dose or discontinuation, when clinically feasible, since the first version of these guidelines was published in 1997 [6–11].

In contrast, renin–angiotensin system (RAS) inhibitors and β-blockers have been recommended in HF with reduced EF (HFrEF) in all versions of the ESC guidelines due to proven prognostic benefits [6–11]. In addition, mineralocorticoid receptor antagonists (MRAs) have been recommended for symptomatic relief in HFrEF since 1997 [6–11], for prognostic benefits in HFrEF with severe symptoms since 2001 [7–11], and for prognostic benefits also in HFrEF with moderate symptoms since 2012 [10, 11]. Thereto, treatment with digitalis has been an option in all versions of the HF guidelines although the prognostic benefits are uncertain [6–11].

The temporal trends in treatment with β-blockers, RAS inhibitors, MRAs, and digitalis during the last decades have been extensively studied in patients with chronic HFrEF [12–17] whereas there is lack of knowledge in the coinciding trends in treatment with loop diuretics in real-life patients with chronic HF. With this background, we aimed to study trends in loop diuretic treatment from 2005 to 2014 in a nationwide cohort of real-life patients with chronic HF. In addition, the coinciding trends for treatment with β-blockers, RAS inhibitors, MRAs, and digitalis in the same cohort were studied.

## Methods

### Registers used in this study and study population inclusion

**Fig. 1:**
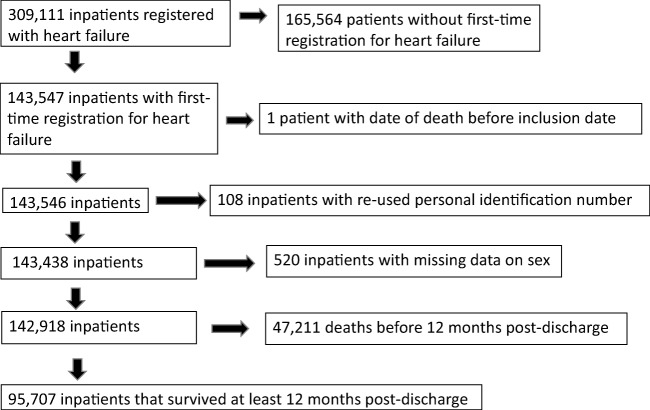
Flow chart of inclusion of patients

In the present study, data from the Swedish National Patient Register (NPR), Swedish Cause of Death register and Swedish Prescribed Drug Register were linked by personal identification codes. NPR contains individual data for all inpatient hospital discharges in Sweden since 1987. These data include primary diagnoses, contributory diagnoses, and admission and discharge dates. More than 99% of all hospitalizations are registered, and the overall validity of the diagnoses is 85–95% [18]. For a primary diagnosis of HF, the validity has been reported as 95% [19]. Diagnoses at discharge were coded using the International Classification of Diseases (ICD) version 10. The discharge codes that applied to HF in this study were I11.0, I13.0, I13.2, I42.0, I42.3–9, I50.0–1, and I50.9. No difference in coding between patients with different types of HF, e.g., HFrEF and HF with preserved EF (HFpEF) exists in ICD version 10. Comorbidity discharge codes used are shown in e-Table 1. A hospital admission registered in the NPR with HF as the primary diagnosis with no previous admission for HF in the past 7 years was defined as a first-time hospitalization for HF. The Swedish Cause of Death register has been in operation since 1961 and includes data on all deaths of people registered in Sweden. We included all patients who survived at least 12 months after discharge from a first-time hospitalization for HF and defined them as patients with chronic HF. The inclusion and exclusion of patients in the present study is shown in Fig. 1. Of the 142,918 patients discharged alive from a first-time hospitalization, 95.707 (67%) survived at least 12 months post-discharge. The end of follow-up was December 31, 2015. From October 1, 2005 to December 31, 2014, 95,707 patients survived at least 12 months after a first-time hospitalization for HF in Sweden. The Swedish Prescribed Drug Register holds records of all dispensed drugs in Sweden since 1999 and since July 2005 with personal identifiers. For drug dispensations, the registration is complete (although demographic data are missing in 0.02–0.6% of cases). The Swedish Prescribed Drug Register has been described previously [20].

The investigation conforms to the principles of the Declaration of Helsinki. The present study was approved by the Regional Ethical Review Board of the University of Gothenburg (540–11, T063–13).

## Statistical analysis

In Sweden, a drug aimed for chronic use in a chronic disease is usually prescribed to last for 3 months before the next dispense. For this reason, we considered a dispensed prescription of any RAS inhibitor, β-blocker (exclusive of sotalol which is mainly used as an anti-arrhythmic in Sweden), MRA, digitalis, loop diuretic, or sinus node I_f_ channel inhibitors during a specified 3-month period as a treatment attempt with the drug during that specified period. The Anatomical Therapeutic Chemical (ATC) codes used in this study are shown in e-Table 2. The proportion of patients treated with each drug class 0–3 months before admission and 0–3, 3–6, 6–9, and 9–12 months after discharge were calculated for each calendar year during the observational period. Temporal trends in proportions between 2005 and 2014 were evaluated with the Cochran–Armitage test for trends in proportions.

Loop diuretics may be used intermittently. Therefore, in addition to trends in the proportion of patients treated with loop diuretics, trends in loop diuretic dose in the patients with prescribed and dispensed loop diuretics were analyzed. The Defined Daily Dose (DDD) is the assumed average maintenance dose per day for a drug class used for its main indication in adults. The DDD was defined by the WHO Collaborating Centre for Drug Statistics Methodology (https://www.whocc.no/ddd/definition_and_general_considera/170314). The DDD for loop diuretics are 40 mg for furosemide, 1 mg for bumetanide, and 15 mg for torasemide. The loop diuretic DDD during a specified period may be evaluated as a marker of overall medicalization with loop diuretics during that period even though day to day changes in dose may not be tracked. The loop diuretic DDD at 0–3 months before admission and 0–3, 3–6, 6–9, and 9–12 months after discharge were calculated for each calendar year during the observational period. We investigated outliers and considered median DDD and interquartile range (IQR) more robust than mean DDD and standard deviation. Temporal trends in the median DDD between 2005 and 2014 were evaluated with quantile regression using the Markov chain marginal bootstrap method.

We considered prescribed and dispensed medication during the 9–12 month post-discharge interval representative of treatment in patients with chronic HF. Thus, our main results are based on the analyses of 9–12 month post-discharge data.

The SAS software version 9.2 (SAS, Cary, NC, USA) and R software version 2 (R Development Core Team, https://www.r-project.org) were used for data analysis. Significance level was set at 0.05.

## Results

### Descriptive data at hospital discharge

Data on sex, age, and comorbidities at discharge from hospital in the study cohort are shown in Table 1. Of the patients, 53.5% were men, 64.2% were aged 75 years or older, 45.8% had ischaemic heart disease, 26.4% had diabetes mellitus, and 58.9% had hypertension at baseline. Annual sex and age distribution from 2005 to 2014 is shown in e-Table 3.

**Table 1:** Sex, age, and comorbidities at hospital discharge in patients that survived at least 12 months after discharge from first-time hospitalization for HF in Sweden in 2005–2014

All patients, *n* (%)	95,707 (100)
Age and sex	
Age, mean (SD), years	76.5 (12.2)
Sex, *n* (%)	
Men	51,118 (53.5)
Women	44,519 (46.5)
Age group (years), *n* (%)	
18–54	5518 (5.8)
55–64	9636 (10.1)
65–74	19,085 (19,9)
75–84	33,086 (35.3)
85–99	27,662 (28.9)
Comorbidities, *n* (%)	
Ischemic heart disease	43,839 (45.8)
Valvular disease	14,956 (15.6)
Stroke	14,882 (15.6)
Peripheral arterial disease	6915 (7.2)
Chronic obstructive pulmonary disease	12,014 (12.6)
Renal failure	9753 (10.2)
Sleep apnea syndrome	2282 (2.4)
Diabetes mellitus	25,274 (26.4)
Obesitas	4802 (5.0)
Hypertension	56,380 (58.9)
Atrial fibrillation	48,157 (50.3)

### Temporal trends in treatment with loop diuretics

**Fig. 2:**
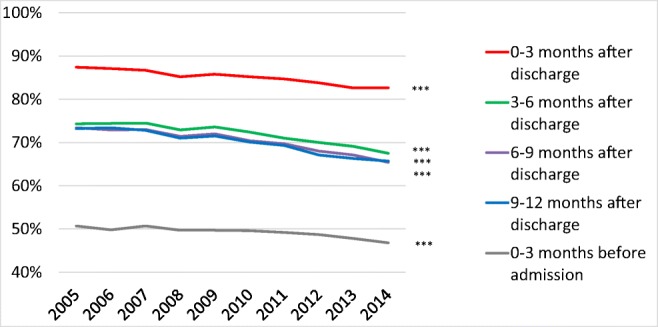
Loop diuretic treatment rates from 2005 to 2014 in patients that survived at least 12 months after discharge after a first-time hospitalization for heart failure in Sweden. ***p < 0.001

The proportion of patients treated with loop diuretics decreased from 2005 to 2014, both before and after a first-time hospitalization for HF (Fig. 2).

The proportion of patients treated with loop diuretics was higher in women than in men, both before and after a first-time hospitalization for HF (Table 2). During the 9–12 months post-discharge period, the proportion of patients treated with loop diuretics decreased from 70.2% in 2005 to 62.6% in 2014 in men and from 77.0% in 2005 to 69.1% in 2014 in women (*p* < 0.001 for trends).

**Table 2:** Loop diuretic treatment rates in patients that survived at least 12 months after discharge from a first-time hospitalization for heart failure in Sweden 2005–2014

Year	2005 Oct–Dec	2006	2007	2008	2009	2010	2011	2012	2013	2014	*p* value for trend
Loop diuretic treatment rate, %											
Month 0–3 before admission											
All	50.7	49.8	50.7	49.7	49.7	49.6	49.2	48.7	47.8	46.8	< 0.001
Men	48.0	45.7	46.5	45.6	46.3	46.0	44.9	44.2	44.1	43.9	< 0.001
Women	54.2	54.5	55.6	54.5	53.5	53.6	54.2	53.9	52.0	50.0	< 0.001
Age 18–54	23.3	23.5	26.4	17.9	17.7	20.0	18.5	20.1	20.8	19.1	0.013
Age 55–64	35.8	33.9	34.4	37.7	34.6	34.7	37.0	33.3	32.6	31.4	0.058
Age 65–74	49.4	47.7	48.7	44.9	45.5	45.2	45.8	45.0	44.6	42.6	< 0.001
Age 75–84	52.2	53.1	54.1	53.6	53.8	53.7	53.7	51.9	52.1	50.6	0.006
Age 85–99	61.7	58.4	60.0	58.8	58.4	57.8	56.5	58.8	54.8	55.8	< 0.001
Month 0–3 after discharge											
All	87.4	87.1	86.7	85.2	85.8	85.2	84.7	83.8	82.6	82.6	< 0.001
Men	86.5	86.0	85.6	84.5	84.4	83.7	83.9	82.2	81.4	82.1	< 0.001
Women	88.6	88.4	87.9	86.0	87.4	86.8	85.6	85.7	84.0	83.2	< 0.001
Age 18–54	74.4	74.6	68.2	66.3	66.6	66.1	66.2	61.1	61.7	63.5	< 0.001
Age 55–64	83.6	79.5	77.8	77.0	74.6	74.2	74.5	72.5	71.3	71.7	< 0.001
Age 65–74	87.1	84.0	85.9	83.0	83.5	82.6	80.4	79.7	78.5	77.8	< 0.001
Age 75–84	87.3	89.3	89.5	88.1	88.4	87.1	88.1	87.1	85.5	85.9	< 0.001
Age 85–99	92.1	91.7	91.2	89.7	91.5	91.5	90.7	91.4	89.6	89.7	< 0.001
Month 3–6 after discharge											
All	74.3	74.4	74.5	72.9	73.6	72.4	71.0	70.0	69.1	67.5	< 0.001
Men	71.1	72.1	72.1	70.1	70.8	69.1	67.8	67.1	67.0	64.7	< 0.001
Women	78.5	77.0	77.4	76.1	76.7	76.2	74.8	73.2	71.4	70.7	< 0.001
Age 18–54	55.8	55.2	52.5	44.9	49.3	48.1	42.4	42.9	43.0	46.7	< 0.001
Age 55–64	62.2	61.5	60.6	61.9	57.5	58.3	54.7	56.0	55.0	53.5	< 0.001
Age 65–74	71.0	69.3	72.1	68.1	68.2	66.2	66.6	63.2	62.7	59.2	< 0.001
Age 75–84	75.9	77.3	78.1	76.1	77.3	74.7	74.9	73.4	72.1	70.0	< 0.001
Age 85–99	83.5	83.0	82.2	81.6	82.6	82.7	80.8	81.1	79.3	79.1	< 0.001
Month 6–9 after discharge											
All	73.4	72.9	73.0	71.4	72.0	70.4	69.7	68.0	67.1	65.4	< 0.001
Men	71.1	70.3	70.9	68.5	68.8	67.4	66.5	64.4	63.9	62.3	< 0.001
Women	76.5	75.9	75.5	74.9	75.6	73.7	73.5	72.1	70.7	68.9	< 0.001
Age 18–54	49.6	54.5	50.2	43.2	39.6	43.9	37.4	39.0	38.8	42.3	< 0.001
Age 55–64	59.9	58.7	59.2	60.0	55.8	53.5	51.8	52.4	50.3	49.0	< 0.001
Age 65–74	70.6	68.4	70.1	64.0	65.1	64.2	63.8	60.3	59.8	56.0	< 0.001
Age 75–84	76.1	75.6	75.9	75.7	75.8	72.9	73.9	71.1	69.8	68.4	< 0.001
Age 85–99	82.4	81.7	82.2	81.0	83.4	81.5	81.4	81.1	79.4	78.5	< 0.001
Month 9–12 after discharge											
All	73.2	73.4	72.8	71.0	71.5	70.1	69.3	67.1	66.3	65.7	< 0.001
Men	70.2	70.2	70.0	67.7	68.1	66.3	66.3	63.2	63.5	62.6	< 0.001
Women	77.0	77.1	76.1	74.9	75.4	74.2	72.9	71.6	69.5	69.1	< 0.001
Age 18–54	51.9	48.6	48.6	37.7	42.0	41.6	35.1	37.0	39.5	39.2	< 0.001
Age 55–64	60.2	59.7	56.9	58.2	54.4	51.7	49.6	47.6	47.8	47.6	< 0.001
Age 65–74	71.7	67.9	69.3	65.1	64.0	63.2	63.2	60.1	59.7	58.4	< 0.001
Age 75–84	74.2	76.6	76.0	74.8	75.2	72.7	73.9	71.0	68.9	69.0	< 0.001
Age 85–99	82.9	83.4	83.2	81.6	83.3	82.3	81.6	80.5	78.6	78.0	< 0.001

The proportion of patients treated with loop diuretics was higher in older patients than in younger patients both before and after a first-time hospitalization for HF (Table 2). During the 9–12 months post-discharge period, the proportion of patients treated with loop diuretics decreased from 51.9% in 2005 to 39.2% in 2014 in patients aged 18–54 years) and from 82.9 to 78.0% in patients aged 85–99 years (*p* < 0.001 for trends).

During the 9–12 months post-discharge period, the median loop diuretic DDD decreased from 2.13 (IQR 1.09–2.77) in 2005 to 1.63 (IQR 1.09–2.25) in 2014 (*p* = 0.005 for trend) (e-Table 4). Trends for decreased median loop diuretic DDD during the 9–12 months post-discharge period were observed in all subgroups.

### Temporal trends in treatment with RAS inhibitors

**Fig. 3:**
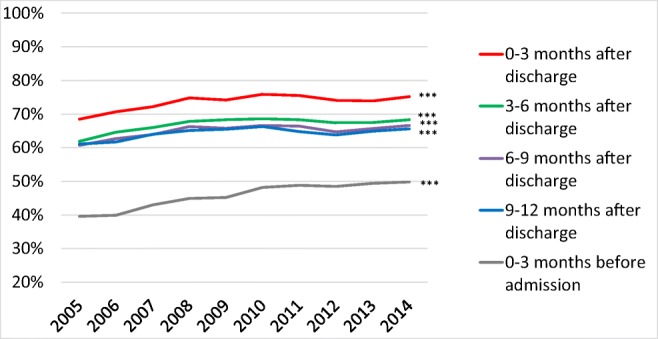
RAS inhibitor treatment rates from 2005 to 2014 in patients that survived at least 12 months after discharge after a first-time hospitalization for heart failure in Sweden. ***p < 0.001

The proportion of patients treated with RAS inhibitors increased from 2005 to 2014, both before and after a first-time hospitalization for HF (Fig. 3) (*p* for trends <0.001).

During the observational period, the proportion of patients treated with RAS inhibitors were higher in men than in women (e-Table 5) but increased more in women than in men. During the 9–12 months post-discharge period, the proportion of male patients treated with RAS inhibitors increased only slightly from 65.7 to 67.5% between 2005 and 2014 (*p* for trend 0.97). Corresponding rates in women were 55.0 and 63.5% (*p* < 0.001 for trend).

The proportion of patients treated with RAS inhibitors post-discharge were higher in younger than in older patients during the observational period (e-Table 5), but increased more in the oldest patients. During the 9–12 months post-discharge period, the use of RAS inhibitors rose from 46.4 to 58.2% in patients aged 85–99 years (*p* for trend < 0.001).

### Temporal trends in treatment with β-blockers

**Fig. 4:**
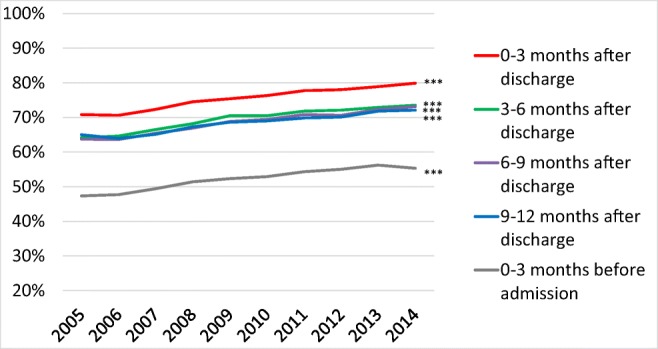
β-blocker treatment rates from 2005 to 2014 in patients that survived at least 12 months after discharge after a first-time hospitalization for heart failure in Sweden. ***p < 0.001

The proportion of patients treated with β-blockers increased from 2005 to 2014, both before and after a first-time hospitalization for HF (Fig. 4) (*p* for trends < 0.001).

During the 9–12 months post-discharge period, treatment with β-blockers rose from 67.5 to 71.6% in men (e-Table 6) and from 61.7 to 72.6% in women (*p* for trends < 0.001).

The proportion of patients treated with β-blockers post-discharge was higher in younger than in older patients (e-Table 6) and increased only slightly among patients aged 18–54 years, from 69.0 to 71.0%, but from 54.4 to 68.2% in patients aged 85–99 years (*p* < 0.001 for trends).

### Temporal trends in treatment with MRAs

**Fig. 5:**
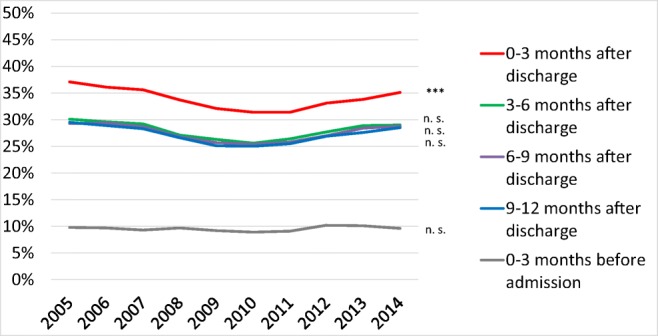
MRA treatment rates from 2005 to 2014 in patients that survived at least 12 months after discharge after a first-time hospitalization for heart failure in Sweden. ***p < 0.001; *n.s.* not significant

The proportion of patients treated with MRAs post-discharge decreased in the beginning and increased in the end of the observational period (Fig. 5).

During the 9–12 months post-discharge period, the MRA treatment increased slightly from 29.2% in 2005 to 30.5% in 2014 in men (*p* = 0.0352 for trend) whereas the corresponding rates in women decreased from 29.9 to 26.1% (*p* < 0.001 for trend) (e-Table 7).

The proportion of patients treated with MRAs was higher in younger patients than in older patients (e-Table 7) and increased in patients aged 18–54 years from 26.4% in 2005 to 39.9% in 2015 but decreased in patients aged 85–99 from 24.7 to 20.7% (*p* < 0.001 for trends).

### Temporal trends in treatment with digitalis

**Fig. 6:**
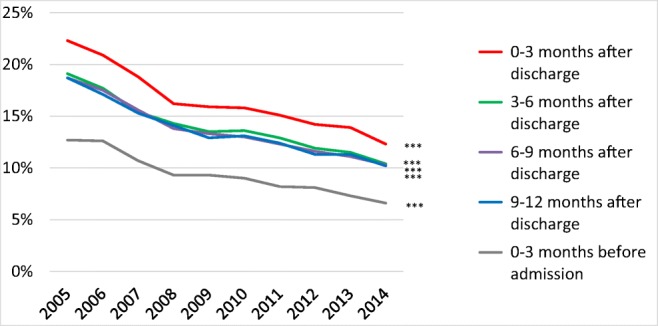
Digitalis treatment rates from 2005 to 2014 in patients that survived at least 12 months after discharge after a first-time hospitalization for heart failure in Sweden. ***p < 0.001

During the observational period, the proportion of patients treated with digitalis decreased (Fig. 6) (*p* < 0.001 for trends). The proportion of patients treated with digitalis was higher in women than in men and in older patients than in younger patients both before and after a first-time hospitalization for HF (e-Table 8).

### Temporal trends in treatment with ivabradine

In our cohort, only 327 prescriptions for ivabradine were dispensed during the entire observational period (data not shown). Therefore, no temporal trends were estimated.

## Discussion

We studied temporal trends for loop diuretic treatment from 2005 to 2014 in 95,707 real-life patients with chronic HF. Our most significant additions to current knowledge were that both treatments with loop diuretics per se and loop diuretic dose decreased. In addition, we observed that treatment with neuro-hormonal antagonists increased and that age- and sex-related differences in β-blocker and RAS inhibitor treatment decreased in this cohort.

### Descriptive data at hospital discharge

The descriptive data in the present study shows the demographic and comorbidity characteristics in a real-life nationwide cohort of patients with chronic HF. A previous study on Swedish patients demonstrated that patients enrolled in a HF registry were more likely of male sex, younger age, less comorbidities, and better utilization of HF medications when compared to real-life Swedish patients with HF [21]. In addition, the demographic and comorbidity characteristics of patients with HFrEF and HFpEF are known to be different. For example, hypertension is more frequent in HFpEF whereas ischemic heart disease is more frequent in HFrEF [22]. Consequently, trends for loop diuretic treatments in selected cohorts may not automatically be generalized to real-life cohorts with HF.

### Temporal trends for pharmacological treatment

The trends for decreased treatment with loop diuretics in the present study of real-life patients with HF were consistent with trends in previous studies of patients with HFrEF [13, 14]. Our study is, to our knowledge, the first where temporal trends on loop diuretic dose were investigated and consequently the first where a trend for decreased loop diuretic dose has been observed. In other observational studies, both loop diuretic treatment per se [1, 2] and higher loop diuretic dose [3–5] have been associated with increased long-term mortality in patients with HF irrespective of their symptomatic severity. Proposed explanatory mechanisms of the association between loop diuretics and increased mortality in HF have been through decreased blood pressure, worsened renal function, neuroendocrine activation, and increased prevalence of arrhythmias [23–26]. However, loop diuretic treatment has also been proposed to be a marker for HF disease severity rather than an independent risk factor for increased mortality [27].

If the decrease in loop diuretic treatment observed during the investigated period in the present study was associated with improved adherence to HF guidelines or with decreased tendency of fluid retention could not be elucidated as we had no data on fluid retention. It also remains unknown if the trends for decreased loop diuretic treatment had any impact on trends in mortality.

The temporal trends for increased RAS inhibitor, increased β-blocker, and decreased digitalis treatment in our study were consistent with trends in patients with chronic HFrEF [12–17]. The temporal trend for decreased treatment with MRAs during the first years of our observational period was consistent with observations in Swedish patients with chronic HFrEF 2003–2012 [17]. However, there was a slight increase in MRA treatment during the final years of our observational period. This increase coincided with the publication of the HF guidelines in 2012 where treatment with MRAs for prognostic benefits in HFrEF was extended from patients with severe symptoms to patients with moderate symptoms [11].

If the observed increase in neuro-hormonal antagonist treatment in our real-life cohort with HF were associated with improved adherence to guidelines on treatment of HFrEF could not be answered by this study due to lack of data on EF. In contrast to in HFrEF, neuro-hormonal antagonists have never been recommended to treat HFpEF due to lack of proven prognostic benefits. Contemporary trends of increased proportion of patients with HFpEF in prevalent HF [28] and increased prevalence of comorbidities frequently treated with neuro-hormonal antagonists, for example hypertension and ischaemic heart disease, in patients with HF [29] have been suggested. These possible trends in EF and comorbidities may have influenced the observed trends in neuro-hormonal antagonists.

We observed lower neuro-hormonal antagonist and β-blocker treatment rates in women and older patients when compared to men and younger patients, respectively. These results are consistent with findings in previous studies on sex- and age-related differences in HF treatment where available data on clinical characteristics and comorbidities could not fully explain the inequities [30, 31]. Sex-related differences in neuro-hormonal antagonist and β-blocker treatment have been shown to remain after adjustment for age [30]. Temporal trends in our study suggest that these sex- and age-related differences in HF treatment may have decreased with time. However, the trends in EF and comorbidities suggested in other studies [28, 29] mentioned above may also have influenced our results on age- and sex-related trends. More studies of these remaining gaps in knowledge are needed.

## Strengths and limitations

Sweden has a universal healthcare system that provides healthcare to the Swedish population. The coverage of the registries used in this study shows that our study cohort is representative of a nationwide cohort of real-life patients with chronic HF. During the observational period, there were no significant changes in coding, reimbursement, prescription, and dispensing systems in Sweden that might have affected our results. However, the number of Swedish hospital beds has decreased (OECD Health Data 2012; Eurostat Statistics Database; WHO European Health for All Database), which may have influenced the inclusion of patients to a limited extent.

We acknowledge the lack of data on EF and symptomatic severity. However, recommendations on loop diuretic treatment in real-life patients with HF have never depended on EF. Nevertheless, the lack of data on symptomatic severity limited the ability of this study to investigate possible trends of improved adherence to guideline recommendations on loop diuretic treatment.

## Conclusion

Loop diuretic treatment, with the lowest clinically possible dose, has been recommended in ESC guidelines since 1997 to treat patients with HF and symptoms of fluid retention. In a nationwide cohort of 95,707 real-life patients with chronic HF, we observed a trend for decreased loop diuretic treatment per se and for decreased loop diuretic dose from 2005 to 2014. The prognostic impact of these findings was not elucidated in this study and remains unclear.

## Electronic supplementary material

ESM 1 (DOCX 61 kb)
