# Supplementary material for: Decrease in loop diuretic treatment from 2005 to 2014 in Swedish real-life patients with chronic heart failure
Source: Eur J Clin Pharmacol. 2018 Oct 15;75(2):247–54. doi: 10.1007/s00228-018-2574-6 (PMC6348069; doi:10.1007/s00228-018-2574-6)
Supplement: Supplementary file 1 — (DOCX 61 kb) [file 228_2018_2574_MOESM1_ESM.docx]

**e-Table 1. ICD-10 codes used for comorbidities.**

| **Comorbidity** | **ICD-10 codes** |
| --- | --- |
| Ischaemic heart disease | I20-I22, I24, I25 |
| Valvular disease | I34-I37 |
| Stroke | I60-I64, I69 |
| Periferial arterial disease | I70, I73.9 |
| Chronic obstructive pulmonary disease | J44 |
| Renal failure | N17.0-N17.2, N17.8-N17.9, N18 |
| Sleep apnoea syndrome | G47.3 |
| Diabetes mellitus | E10, E11, E12, E13, E14 |
| Obesitas | E66.0-E66.2, E66.8-E66.9 |
| Hypertension | I10, I11.9, I12.0-I12.9, I13.1-I13 |
| Atrial fibrillation | I48 |

**e-Table 2. ATC codes used for treatments.**

|  | **ATC** | | **Drug** | | | **DDD** | | | **unit** | |
| --- | --- | --- | --- | --- | --- | --- | --- | --- | --- | --- |
| **RAS inhibitors** | | | | | | | | | | |
|  | **C09AA01** | | **captopril** | | | **50** | | | **mg** | |
|  | **C09AA02** | | **enalapril** | | | **10** | | | **mg** | |
|  | **C09AA03** | | **lisinopril** | | | **10** | | | **mg** | |
|  | **C09AA04** | | **perindopril** | | | **4** | | | **mg** | |
|  | **C09AA05** | | **ramipril** | | | **2.5** | | | **mg** | |
|  | **C09AA09** | | **fosinopril** | | | **15** | | | **mg** | |
|  | **C09BA02** | | **enalapril and diuretics** | | | **10** | | | **mg** | |
|  | **C09BA03** | | **lisinopril and diuretics** | | | **10** | | | **mg** | |
|  | **C09BA05** | | **ramipril and diuretics** | | | **2.5** | | | **mg** | |
|  | **C09BA06** | | **quinapril and diuretics** | | | **15** | | | **mg** | |
|  | **C09CA01** | | **losartan** | | | **50** | | | **mg** | |
|  | **C09CA02** | | **eprosartan** | | | **0.6** | | | **g** | |
|  | **C09CA03** | | **valsartan** | | | **80** | | | **mg** | |
|  | **C09CA04** | | **irbesartan** | | | **0.15** | | | **g** | |
|  | **C09CA06** | | **candesartan** | | | **8** | | | **mg** | |
|  | **C09CA07** | | **telmisartan** | | | **40** | | | **mg** | |
|  | **C09DA01** | | **losartan and diuretics** | | | **50** | | | **mg** | |
|  | **C09DA02** | | **eprosartan and diuretics** | | | **0.6** | | | **mg** | |
|  | **C09DA03** | | **valsartan and diuretics** | | | **80** | | | **mg** | |
|  | **C09DA04** | | **irbesartan and diuretics** | | | **0.15** | | | **mg** | |
|  | **C09DA06** | | **candesartan and diuretics** | | | **8** | | | **mg** | |
|  | **C09DA07** | | **telmisartan and diuretics** | | | **40** | | | **mg** | |
|  | **C09DB01** | | **valsartan and amlodipine** | | | **80** | | | **mg** | |
| **Β-blockers** | | | | | | | | | | |
|  | **C07AB02** | **metoprolol** | | | | | **0.15** | | | **g** |
|  | **C07AB07** | **bisoprolol** | | | | | **10** | | | **mg** |
|  | **C07AG02** | **carvedilol** | | | | | **37.5** | | | **mg** |
|  | **C07AB03** | **atenolol** | | | | | **75** | | | **mg** |
|  | **C07FB02** | **metoprolol and felodipine** | | | | | **0,15** | | | **g** |
| **MRA** | | | | | | | | | | |
|  | **C03DA01** | | | **spironolactone** | **75** | | | **mg** | | |
|  | **C03DA04** | | | **eplerenone** | **50** | | | **mg** | | |
| **Digitalis** | | | | | | | | | | |
|  | **C01AA04** | | | **digitoxin** | **0.1** | | | **mg** | | |
|  | **C01AA05** | | | **digoxin** | **0.25** | | | **mg** | | |
| **Loop diuretics** | | | | | | | | | | |
|  | **C03CA01** | | | **furosemide** | **40** | | | **mg** | | |
|  | **C03CA02** | | | **bumetanide** | **1** | | | **mg** | | |
|  | **C03CA04** | | | **torasemide** | **15** | | | **mg** | | |
| **I_f_ channel inhibitor** | | | | | | | | | | |
|  | **C01EB17** | | | **ivabradine** | **10** | | | **mg** | | |

**e-Table 3. Sex and age distribution in patients that survived at least 12 months after discharge from a first-time hospitalization for heart failure in Sweden 2005–2014.**

| **Year** | **2005 Oct-Dec** | **2006** | **2007** | **2008** | **2009** | **2010** | **2011** | **2012** | **2013** | **2014** |
| --- | --- | --- | --- | --- | --- | --- | --- | --- | --- | --- |
| **Number of patients (%)** |  |  |  |  |  |  |  |  |  |  |
| **All** | 2,483 (100) | 10,049 (100) | 10,200 (100) | 10,342 (100) | 10,441 (100) | 10,552 (100) | 10,631 (100) | 10,562 (100) | 10,399 (100) | 10,048 (100) |
| **Men** | 1,400 (56.4) | 5,424 (54.0) | 5,472 (53.6) | 5,593 (54.1) | 5,526 (52.9) | 5,542 (52.5) | 5,713 (53.7) | 5,632 (53.3) | 5,540 (53.3) | 5,346 (53.2) |
| **Women** | 1,083 (43.6) | 4,625 (46.0) | 4,728 (46.4) | 4,749 (45.9) | 4,915 (47.1) | 5,010 (47.5) | 4,918 (46.3) | 4,930 (46.7) | 4,859 (46.7) | 4,702 (46.8) |
| **Aged 18-54** | 129 (5.2) | 587 (5.8) | 625 (6.1) | 570 (5.5) | 560 (5.4) | 599 (5.7) | 621 (5.8) | 638 (6.0) | 572 (5.5) | 617 (6.1) |
| **Aged 55-64** | 299 (12.0) | 1,045 (10.4) | 1,141 (11.2) | 1,076 (10.4) | 1,072 (10.3) | 1,020 (9.7) | 1,028 (9.7) | 1,045 (9.9) | 985  (9.5) | 925 (9.2) |
| **Aged 65-74** | 466 (18.8) | 1,945 (19.4) | 1,967 (19.3) | 2,016 (19.5) | 1,991 (19.1) | 2,010 (19.0) | 2,168 (20.4) | 2,229 (21.1) | 2,161 (20.8) | 2,132 (21.2) |
| **Aged 75-84** | 929 (37.4) | 3,752 (37.3) | 3,715 (36.4) | 3,818 (36.9) | 3,753 (35.9) | 3,667 (34.8) | 3,737 (35.2) | 3,521 (33.3) | 3,554 (34.2) | 3,360 (33.4) |
| **Aged 85-99** | 660 (26.6) | 2,720 (27.1) | 2,752 (27.0) | 2,862 (27.7) | 3,065 (29.4) | 3,256 (30.9) | 3,077 (28.9) | 3,129 (29.6) | 3,127 (30.1) | 3,014 (30.0) |

**e-Table 4, Loop diuretic doses in patients in patients that survived at least 12 months after discharge from a first-time hospitalization for heart failure in Sweden 2005–2014.**

| **Year** | **2005 Oct-Dec** | **2006** | **2007** | **2008** | **2009** | **2010** | **2011** | **2012** | **2013** | **2014** | **P-value**  **for trend** |
| --- | --- | --- | --- | --- | --- | --- | --- | --- | --- | --- | --- |
| **Defined Daily Dose^a^, 0-3 months before admission, median (IQR)** | | | | | | | | | | | |
| **All** | 1.10  (1.07- 2.17) | 1.15  (1.09- 2.20) | 1.12  (1.09- 2.19) | 1.10  (1.07- 2.17) | 1.11  (1.07- 2.17) | 1.11  (1.07- 2.17) | 1.11  (1.07- 2.17) | 1.10  (1.07- 2.17) | 1.11  (1.07- 2.17) | 1.11  (1.07- 2.17) | 0.411 |
| **Men** | 1.10  (1.08- 2.20) | 1.14  (1.09- 2.20) | 1.12  (1.09- 2.20) | 1.10  (1.07- 2.18) | 1.11  (1.07- 2.18) | 1.11  (1.08- 2.17) | 1.11  (1.08- 2.17) | 1.10  (1.09- 2.18) | 1.11  (1.09- 2.17) | 1.11  (1.09- 2.17) | 0.456 |
| **Women** | 1.10  (1.07- 2.17) | 1.15  (1.07- 2.17) | 1.12  (1.07- 2.17) | 1.10  (1.07- 2.17) | 1.11  (1.07- 2.17) | 1.11  (0.99-2.17) | 1.11  (1.07- 2.17) | 1.10  (1.07- 2.17) | 1.10  (1.05- 2.17) | 1.11  (1.07- 2.17) | 0.278 |
| **Age 18-54** | 1.11  (1.09- 2.93) | 1.11  (1.07- 2.17) | 1.11  (1.07- 2.20) | 1.10  (1.07- 2.17) | 1.09  (0.84- 2.20) | 1.09  (0.99-2.17) | 1.11  (0.82- 2.17) | 1.10  (0.91- 2.17) | 1.10  (1.09- 2.22) | 1.09  (1.07- 2.09) | 0.059 |
| **Age 55-64** | 1.10  (1.09- 2.20) | 1.12  (1.08- 2.20) | 1.11  (1.09- 2.22) | 1.11  (1.08- 2.20) | 1.11  (1.08- 2.20) | 1.11  (1.07- 2.20) | 1.10  (1.08- 2.17) | 1.10  (1.09- 2.17) | 1.11  (1.09- 2.17) | 1.11  (1.09- 2.17) | 0.624 |
| **Age 65-74** | 1.61  (1.08- 2.20) | 1.12  (1.09- 2.20) | 1.12  (1.09- 2.22) | 1.11  (1.08- 2.17) | 1.11  (1.09- 2.18) | 1.11  (1.09- 2.18) | 1.12  (1.09- 2.20) | 1.10  (1.09- 2.20) | 1.11  (1.09- 2.17) | 1.11  (1.09- 2.17) | 0.106 |
| **Age 75-84** | 1.10  (1.09- 2.17) | 1.12  (1.09- 2.17) | 1.36  (1.09- 2.20) | 1.11  (1.07- 2.18) | 1.11  (1.07- 2.17) | 1.11  (1.07- 2.17) | 1.11  (1.09- 2.17) | 1.10  (1.08- 2.17) | 1.11  (1.09- 2.17) | 1.11  (1.09- 2.17) | 0.406 |
| **Age 85-99** | 1.10  (0.91- 2.17) | 1.38  (1.07- 2.20) | 1.12  (1.00- 2.17) | 1.10  (0.95- 2.17) | 1.11  (0.93- 2.17) | 1.11  (0.93-2.17) | 1.11  (0.93- 2.15) | 1.10  (0.92- 2.13) | 1.10  (0.88- 2.13) | 1.10  (0.91- 2.15) | 0.212 |
| **Defined Daily Dose, 0-3 months after discharge, median (IQR)** | | | | | | | | | | | |
| **All** | 2.17  (1.11- 3.26) | 2.17  (1.10- 3.24) | 2.17  (1.10- 3.23) | 2.15  (1.10- 2.84) | 2.15  (1.10- 2.80) | 2.16  (1.10- 2.76) | 2.17  (1.10- 2.78) | 2.13  (1.09- 2.72) | 2.10  (1.09- 2.70) | 2.13  (1.09- 2.72) | 0.008 |
| **Men** | 2.17  (1.11- 3.26) | 2.17  (1.10- 3.26) | 2.17  (1.10- 3.26) | 2.17  (1.10- 2.99) | 2.15  (1.10- 3.09) | 2.17  (1.10- 2.81) | 2.17  (1.10- 2.95) | 2.15  (1.09- 2.72) | 2.17  (1.09- 2.72) | 2.15  (1.09- 2.72) | 0.147 |
| **Women** | 2.17  (1.11- 2.78) | 2.17  (1.10- 3.10) | 2.17  (1.10- 3.13) | 2.15  (1.10- 2.78) | 2.09  (1.10- 2.75) | 2.07  (1.10- 2.72) | 2.09  (1.10- 2.72) | 1.92  (1.09- 2.67) | 1.87  (1.09- 2.46) | 1.97  (1.09- 2.52) | <0.001 |
| **Age 18-54** | 1.67  (1.11- 2.22) | 1.74  (1.09- 2.45) | 2.16  (1.09- 2.72) | 1.87  (1.09- 2.20) | 2.15  (1.09- 2.72) | 1.63  (1.09- 2.20) | 1.63  (1.09- 2.33) | 1.65  (1.09- 2.22) | 1.67  (1.09- 2.22) | 1.90  (1.09- 2.25) | 0.579 |
| **Age 55-64** | 2.17  (1.11- 2.78) | 2.17  (1.09- 3.10) | 2.17  (1.10- 3.26) | 2.15  (1.10- 2.96) | 2.15  (1.09- 2.78) | 2.17  (1.10- 2.84) | 2.13  (1.09- 2.72) | 2.15  (1.09- 2.59) | 1.69  (1.09- 2.36) | 2.17  (1.09- 2.75) | 0.211 |
| **Age 65-74** | 2.17  (1.11- 3.33) | 2.17  (1.10- 3.26) | 2.17  (1.10- 3.26) | 2.17  (1.10- 3.24) | 2.15  (1.09- 2.91) | 2.17  (1.09- 3.22) | 2.17  (1.10- 3.26) | 2.17  (1.09- 3.12) | 2.17  (1.09- 2.72) | 2.17  (1.09- 2.78) | 0.874 |
| **Age 75-84** | 2.17  (1.11- 3.26) | 2.17  (1.11- 3.26) | 2.17  (1.10- 3.26) | 2.17  (1.10- 3.03) | 2.15  (1.10- 2.98) | 2.17  (1.10- 2.88) | 2.17  (1.10- 2.80) | 2.15  (1.09- 2.72) | 2.17  (1.09- 2.72) | 2.15  (1.09- 2.72) | 0.146 |
| **Age 85-99** | 2.17  (1.11- 2.91) | 2.17  (1.11- 3.22) | 2.17  (1.10- 3.00) | 2.13  (1.10- 2.75) | 2.13  (1.10- 2.75) | 1.91  (1.10- 2.72) | 1.97  (1.10- 2.72) | 1.90  (1.09- 2.50) | 1.90  (1.09- 2.45) | 1.86  (1.09- 2.40) | <0.001 |
| **Defined Daily Dose, 3-6 months after discharge, median (IQR)** | | | | | | | | | | | |
| **All** | 1.83  (1.10- 2.66) | 1.83  (1.09- 2.72) | 1.83  (1.09- 2.72) | 1.67  (1.09- 2.50) | 1.65  (1.09- 2.45) | 1.63  (1.09- 2.28) | 1.63  (1.09- 2.39) | 1.63  (1.09- 2.25) | 1.63  (1.09- 2.22) | 1.48  (1.09- 2.22) | <0.001 |
| **Men** | 1.98  (1.11- 2.81) | 1.87  (1.09- 2.72) | 1.83  (1.09- 2.75) | 1.67  (1.09- 2.67) | 1.66  (1.09- 2.70) | 1.65  (1.09- 2.47) | 1.63  (1.09- 2.47) | 1.63  (1.09- 2.25) | 1.63  (1.09- 2.25) | 1.60  (1.09- 2.25) | <0.001 |
| **Women** | 1.69  (1.09- 2.30) | 1.83  (1.09- 2.52) | 1.82  (1.09- 2.72) | 1.65  (1.09- 2.45) | 1.63  (1.09- 2.33) | 1.63  (1.09- 2.25) | 1.63  (1.09- 2.25) | 1.63  (1.09- 2.22) | 1.54  (1.09- 2.22) | 1.40  (1.09- 2.20) | 0.003 |
| **Age 18-54** | 1.69  (1.09- 2.22) | 1.11  (1.09- 2.20) | 1.63  (1.09- 2.20) | 1.12  (1.09- 2.22) | 1.11  (1.09- 2.17) | 1.11  (1.09- 2.17) | 1.10  (1.09- 2.20) | 1.11  (1.09- 2.18) | 1.11  (1.09- 2.20) | 1.12  (1.09- 2.17) | 0.058 |
| **Age 55-64** | 2.17  (1.11- 2.90) | 1.63  (1.09- 2.45) | 1.63  (1.09- 2.45) | 1.63  (1.09- 2.72) | 1.65  (1.09- 2.25) | 1.63  (1.09- 2.45) | 1.11  (1.09- 2.20) | 1.12  (1.09- 2.22) | 1.12  (1.09- 2.22) | 1.12  (1.09- 2.70) | <0.001 |
| **Age 65-74** | 1.69  (1.11- 2.58) | 1.90  (1.09- 2.75) | 1.83  (1.09- 2.99) | 1.67  (1.09- 2.65) | 1.63  (1.09- 2.72) | 1.65  (1.09- 2.72) | 1.65  (1.09- 2.72) | 1.65  (1.09- 2.72) | 1.63  (1.09- 2.25) | 1.63  (1.09- 2.70) | 0.043 |
| **Age 75-84** | 1.83  (1.11- 2.52) | 1.90  (1.09- 2.72) | 1.85  (1.09- 2.75) | 1.73  (1.09- 2.69) | 1.67  (1.09- 2.72) | 1.71  (1.09- 2.72) | 1.65  (1.09- 2.45) | 1.63  (1.09- 2.25) | 1.63  (1.09- 2.25) | 1.61  (1.09- 2.23) | <0.001 |
| **Defined Daily Dose, 6-9 months after discharge, median (IQR)** | | | | | | | | | | | |
| **All** | 1.85  (1.09- 2.75) | 1.77  (1.09- 2.72) | 1.83  (1.09- 2.72) | 1.65  (1.09- 2.66) | 1.65  (1.09- 2.71) | 1.63  (1.09- 2.36) | 1.63  (1.09- 2.45) | 1.63  (1.09- 2.25) | 1.62  (1.09- 2.22) | 1.60  (1.09- 2.22) | 0.001 |
| **Men** | 2.13  (1.09- 3.00) | 1.83  (1.09- 2.72) | 1.84  (1.09- 2.74) | 1.67  (1.09- 2.72) | 1.67  (1.09- 2.72) | 1.65  (1.09- 2.66) | 1.65  (1.09- 2.72) | 1.63  (1.09- 2.34) | 1.63  (1.09- 2.31) | 1.60  (1.09- 2.22) | 0.004 |
| **Women** | 1.65  (1.09- 2.66) | 1.69  (1.09- 2.47) | 1.68  (1.09- 2.72) | 1.65  (1.09- 2.58) | 1.63  (1.09- 2.45) | 1.63  (1.09- 2.22) | 1.63  (1.09- 2.22) | 1.63  (1.09- 2.25) | 1.40  (1.09- 2.22) | 1.58  (1.08- 2.22) | 0.041 |
| **Age 18-54** | 1.63  (1.09- 2.20) | 1.11  (1.09- 2.21) | 1.42  (1.09- 2.20) | 1.63  (1.09- 2.22) | 1.12  (1.09- 2.22) | 1.10  (1.09- 2.17) | 1.10  (1.09- 2.20) | 1.11  (1.09- 2.20) | 1.11  (1.09- 2.22) | 1.11  (1.09- 2.20) | 0.047 |
| **Age 55-64** | 1.98  (1.09- 3.26) | 1.63  (1.09- 2.50) | 1.63  (1.09- 2.72) | 1.64  (1.09- 2.45) | 1.63  (1.09- 2.72) | 1.63  (1.09- 2.29) | 1.63  (1.09- 2.72) | 1.63  (1.09- 2.25) | 1.11  (1.09- 2.22) | 1.12  (1.09- 2.22) | 0.005 |
| **Age 65-74** | 1.83  (1.09- 2.75) | 1.67  (1.09- 2.72) | 1.67  (1.09- 2.75) | 1.67  (1.09- 2.72) | 1.63  (1.09- 2.72) | 1.63  (1.09- 2.72) | 1.72  (1.09- 2.75) | 1.63  (1.09- 2.72) | 1.65  (1.09- 2.63) | 1.63  (1.09- 2.72) | 0.080 |
| **Age 75-84** | 1.83  (1.09- 2.74) | 1.87  (1.09- 2.72) | 1.90  (1.09- 2.75) | 1.67  (1.09- 2.72) | 1.67  (1.09- 2.72) | 1.70  (1.09- 2.66) | 1.65  (1.09- 2.45) | 1.63  (1.09- 2.45) | 1.63  (1.09- 2.28) | 1.63  (1.09- 2.25) | 0.003 |
| **Age 85-99** | 1.85  (1.09- 2.77) | 1.83  (1.09- 2.72) | 1.83  (1.09- 2.61) | 1.63  (1.09- 2.43) | 1.67  (1.09- 2.45) | 1.63  (1.09- 2.22) | 1.63  (1.08- 2.20) | 1.62  (1.09- 2.22) | 1.47  (1.09- 2.20) | 1.48  (1.07- 2.20) | <0.001 |
| **Defined Daily Dose, 9-12 months after discharge, median (IQR)** | | | | | | | | | | | |
| **All** | 2.13  (1.09- 2.77) | 1.83  (1.09- 2.72) | 1.85  (1.09- 2.74) | 1.69  (1.09- 2.72) | 1.67  (1.09- 2.72) | 1.65  (1.09- 2.50) | 1.65  (1.09- 2.47) | 1.63  (1.09- 2.43) | 1.63  (1.09- 2.25) | 1.63  (1.09- 2.25) | 0.005 |
| **Men** | 2.17  (1.09- 3.23) | 1.91  (1.09- 2.78) | 1.98  (1.09- 2.75) | 1.83  (1.09- 2.72) | 1.83  (1.09- 2.75) | 1.83  (1.09- 2.72) | 1.67  (1.09- 2.72) | 1.65  (1.09- 2.72) | 1.65  (1.09- 2.33) | 1.65  (1.09- 2.38) | <0.001 |
| **Women** | 1.83  (1.09- 2.72) | 1.83  ( 1.09-2.66) | 1.78  (1.09- 2.72) | 1.67  (1.09- 2.70) | 1.65  (1.09- 2.45) | 1.63  (1.09- 2.36) | 1.63  (1.09- 2.22) | 1.63  (1.09- 2.25) | 1.63  (1.09- 2.22) | 1.53  (1.08- 2.22) | <0.001 |
| **Age 18-54** | 1.63  (1.09- 2.17) | 1.15  (1.09- 2.22) | 1.60  (1.09- 2.73) | 1.63  (1.09- 2.22) | 1.11  (1.09- 2.20) | 1.11  (1.09- 2.18) | 1.11  (1.09- 2.22) | 1.11  (1.09- 2.22) | 1.11  (1.09- 2.22) | 1.11  (1.09- 2.22) | 0.032 |
| **Age 55-64** | 2.11  (1.09- 2.75) | 1.83  (1.09- 2.72) | 1.63  (1.09- 2.75) | 1.84  (1.09- 2.72) | 1.60  (1.09- 2.72) | 1.65  (1.09- 2.72) | 1.63  (1.09- 2.67) | 1.12  (1.09- 2.20) | 1.40  (1.09- 2.28) | 1.12  (1.09- 2.25) | <0.001 |
| **Age 65-74** | 2.17  (1.09- 3.23) | 2.07  (1.09- 2.85) | 1.87  (1.09- 2.75) | 1.69  (1.09- 2.72) | 1.65  (1.09- 2.72) | 1.64  (1.09- 2.72) | 1.90  (1.09- 2.72) | 1.67  (1.09- 2.78) | 1.75  (1.09- 2.51) | 1.67  (1.09- 2.72) | 0.028 |
| **Age 75-84** | 1.85  (1.09- 2.72) | 1.83  (1.09- 2.72) | 1.90  (1.09- 2.75) | 1.83  (1.09- 2.74) | 1.83  (1.09- 2.75) | 1.83  (1.09- 2.72) | 1.83  (1.09- 2.47) | 1.65  (1.09- 2.43) | 1.63  (1.09- 2.25) | 1.65  (1.09- 2.26) | 0.003 |
| **Age 85-99** | 2.13  (1.09- 3.20) | 1.85  (1.09- 2.74) | 1.83  (1.09- 2.72) | 1.65  (1.09- 2.45) | 1.69  (1.09- 2.58) | 1.63  (1.08- 2.33) | 1.63  (1.08- 2.42) | 1.63  (1.09- 2.25) | 1.63  (1.09- 2.22) | 1.60  (1.07- 2.20) | 0.004 |

**^a^ DDD is calculated by dividing the daily dose of furosemide by 40 mg, the daily dose of bumetanide by 1 mg, and the daily dose of torasemide by 15mg**

**e-Table 5. Renin–angiotensin system inhibitor treatment rates in patients that survived at least 12 months after discharge from a first-time hospitalization for heart failure in Sweden 2005–2014.**

| **Year** | **2005 Oct-Dec** | **2006** | **2007** | **2008** | **2009** | **2010** | **2011** | **2012** | **2013** | **2014** | **P-value for trend** |  |
| --- | --- | --- | --- | --- | --- | --- | --- | --- | --- | --- | --- | --- |
| **RAS treatment rate, %** |  |  |  |  |  |  |  |  |  |  |  |  |
| **Month 0-3 before admission** |  |  |  |  |  |  |  |  |  |  |  |  |
| **All** | 39.6 | 39.9 | 43.0 | 44.9 | 45.2 | 48.2 | 48.8 | 48.5 | 49.4 | 49.8 | <0.001 |  |
| **Men** | 40.4 | 42.2 | 45.1 | 47.3 | 46.8 | 48.9 | 49.9 | 49.6 | 51.2 | 50.6 | <0.001 |  |
| **Women** | 38.6 | 37.3 | 40.6 | 42.2 | 43.3 | 47.5 | 47.6 | 47.2 | 47.5 | 48.8 | <0.001 |  |
| **Age 18-54** | 26.4 | 29.1 | 32.0 | 28.9 | 27.5 | 28.9 | 30.1 | 27.7 | 30.2 | 29.3 | 0.899 |  |
| **Age 55-64** | 39.1 | 39.5 | 40.8 | 43.1 | 44.2 | 44.5 | 48.3 | 44.8 | 46.3 | 42.3 | <0.001 |  |
| **Age 65-74** | 52.8 | 47.1 | 49.5 | 50.5 | 51.3 | 52.5 | 53.0 | 52.3 | 53.2 | 53.9 | <0.001 |  |
| **Age 75-84** | 41.7 | 43.3 | 47.9 | 50.4 | 48.6 | 53.7 | 54.3 | 53.6 | 55.6 | 54.2 | <0.001 |  |
| **Age 85-99** | 30.3 | 32.6 | 35.1 | 37.5 | 40.5 | 44.0 | 43.1 | 45.6 | 44.4 | 48.4 | <0.001 |  |
| **Month 0-3 after discharge** |  |  |  |  |  |  |  |  |  |  |  |  |
| **All** | 68.5 | 70.7 | 72.2 | 74.8 | 74.2 | 75.9 | 75.5 | 74.1 | 73.9 | 75.2 | <0.001 |  |
| **Men** | 73.4 | 75.8 | 76.1 | 78.4 | 77.8 | 78.8 | 78.2 | 76.6 | 76.6 | 77.4 | 0.044 |  |
| **Women** | 62.0 | 64.7 | 67.7 | 70.5 | 70.1 | 72.6 | 72.3 | 71.2 | 70.7 | 72.6 | <0.001 |  |
| **Age 18-54** | 83.7 | 82.3 | 83.7 | 86.8 | 85.9 | 87.0 | 84.5 | 82.3 | 84.6 | 81.7 | 0.416 |  |
| **Age 55-64** | 86.0 | 84.1 | 85.6 | 85.8 | 85.6 | 83.9 | 87.4 | 86.3 | 84.8 | 84.6 | 0.832 |  |
| **Age 65-74** | 78.5 | 79.4 | 79.9 | 83.1 | 82.9 | 82.9 | 81.4 | 80.8 | 81.1 | 81.6 | 0.179 |  |
| **Age 75-84** | 67.7 | 72.4 | 73.8 | 75.6 | 75.0 | 77.3 | 77.1 | 74.4 | 75.3 | 75.8 | <0.001 |  |
| **Age 85-99** | 51.5 | 54.5 | 56.5 | 61.3 | 61.4 | 65.3 | 63.6 | 63.2 | 61.8 | 65.7 | <0.001 |  |
| **Month 3-6 after discharge** |  |  |  |  |  |  |  |  |  |  |  |  |
| **All** | 61.9 | 64.6 | 66.0 | 67.8 | 68.3 | 68.6 | 68.3 | 67.4 | 67.5 | 68.3 | <0.001 |  |
| **Men** | 64.9 | 69.4 | 69.1 | 70.4 | 71.4 | 71.1 | 70.5 | 69.9 | 69.6 | 70.4 | 0.042 |  |
| **Women** | 58.1 | 58.9 | 62.3 | 64.7 | 64.7 | 65.9 | 65.8 | 64.6 | 65.2 | 65.8 | <0.001 |  |
| **Age 18-54** | 73.6 | 75.0 | 73.4 | 76.5 | 77.5 | 76.5 | 77.3 | 75.7 | 72.7 | 75.7 | 0.889 |  |
| **Age 55-64** | 74.9 | 76.5 | 78.4 | 77.3 | 80.1 | 76.9 | 76.8 | 76.7 | 77.5 | 77.6 | 0.936 |  |
| **Age 65-74** | 71.5 | 74.6 | 74.3 | 76.1 | 77.0 | 73.8 | 73.9 | 73.6 | 74.7 | 73.7 | 0.355 |  |
| **Age 75-84** | 61.1 | 66.0 | 67.7 | 68.4 | 68.0 | 70.2 | 69.9 | 68.1 | 68.8 | 68.8 | <0.001 |  |
| **Age 85-99** | 48.2 | 48.5 | 50.8 | 55.7 | 57.0 | 59.5 | 57.8 | 57.4 | 57.1 | 59.5 | <0.001 |  |
| **Month 6-9 after discharge** |  |  |  |  |  |  |  |  |  |  |  |  |
| **All** | 60.7 | 62.7 | 63.9 | 66.3 | 65.8 | 66.6 | 66.4 | 64.7 | 65.7 | 66.6 | <0.001 |  |
| **Men** | 65.6 | 67.3 | 67.2 | 69.2 | 68.5 | 68.8 | 68.2 | 67.2 | 67.7 | 68.9 | 0.198 |  |
| **Women** | 54.5 | 57.4 | 60.1 | 62.8 | 62.8 | 64.3 | 64.3 | 61.9 | 63.4 | 64.0 | <0.001 |  |
| **Age 18-54** | 69.0 | 71.9 | 75.5 | 75.4 | 72.7 | 75.5 | 72.9 | 69.4 | 70.1 | 72.4 | 0.136 |  |
| **Age 55-64** | 75.3 | 72.4 | 74.8 | 76.0 | 76.3 | 73.7 | 74.6 | 74.4 | 76.1 | 76.1 | 0.246 |  |
| **Age 65-74** | 69.1 | 71.3 | 72.4 | 72.2 | 73.3 | 73.0 | 72.8 | 70.9 | 72.4 | 70.4 | 0.619 |  |
| **Age 75-84** | 60.5 | 64.5 | 65.2 | 68.3 | 65.8 | 67.6 | 67.6 | 66.3 | 66.6 | 66.5 | 0.006 |  |
| **Age 85-99** | 47.0 | 48.5 | 48.9 | 53.8 | 56.0 | 57.7 | 56.3 | 54.4 | 55.9 | 59.9 | <0.001 |  |
| **Month 9-12 after discharge** |  |  |  |  |  |  |  |  |  |  |  |  |
| **All** | 61.1 | 61.7 | 64.0 | 65.1 | 65.5 | 66.3 | 64.8 | 63.8 | 64.9 | 65.6 | <0.001 |  |
| **Men** | 65.7 | 66.6 | 67.7 | 67.9 | 68.2 | 68.5 | 67.1 | 66.8 | 67.0 | 67.5 | 0.972 |  |
| **Women** | 55.0 | 56.0 | 59.8 | 61.8 | 62.5 | 63.9 | 62.1 | 60.3 | 62.5 | 63.5 | <0.001 |  |
| **Age 18-54** | 72.1 | 70.4 | 71.4 | 69.5 | 72.0 | 72.8 | 68.9 | 69.7 | 68.7 | 70.2 | 0.388 |  |
| **Age 55-64** | 73.6 | 73.2 | 74.8 | 75.4 | 77.8 | 75.2 | 74.1 | 72.8 | 74.3 | 74.4 | 0.705 |  |
| **Age 65-74** | 73.2 | 70.2 | 71.8 | 73.6 | 72.8 | 71.7 | 71.3 | 70.0 | 72.6 | 70.4 | 0.323 |  |
| **Age 75-84** | 59.8 | 63.2 | 66.3 | 65.9 | 65.6 | 68.0 | 66.3 | 65.2 | 66.1 | 66.1 | 0.005 |  |
| **Age 85-99** | 46.4 | 47.5 | 49.3 | 53.2 | 55.2 | 57.1 | 54.3 | 53.5 | 54.6 | 58.2 | <0.001 |  |

RAS, renin-angiotensin system.

**e-Table 6. β-blocker treatment rates in patients that survived at least 12 months after discharge from a first-time hospitalization for heart failure in Sweden 2005–2014.**

| **Year** | **2005 Oct-Dec** | **2006** | **2007** | **2008** | **2009** | **2010** | **2011** | **2012** | **2013** | **2014** | **P-value for trend** |
| --- | --- | --- | --- | --- | --- | --- | --- | --- | --- | --- | --- |
| **Β-blocker treatment rate, %** |  |  |  |  |  |  |  |  |  |  |  |
| **Month 0-3 before admission** |  |  |  |  |  |  |  |  |  |  |  |
| **All** | 47.3 | 47.7 | 49.4 | 51.4 | 52.3 | 52.9 | 54.3 | 55.0 | 56.2 | 55.3 | <0.001 |
| **Men** | 46.7 | 47.0 | 48.0 | 49.8 | 50.9 | 50.3 | 52.5 | 52.5 | 54.6 | 53.6 | <0.001 |
| **Women** | 48.0 | 48.6 | 51.1 | 53.3 | 53.8 | 55.8 | 56.5 | 57.7 | 58.1 | 57.3 | <0.001 |
| **Age 18-54** | 30.2 | 28.6 | 32.6 | 26.7 | 29.8 | 31.9 | 30.8 | 33.5 | 32.0 | 27.9 | 0.551 |
| **Age 55-64** | 45.8 | 42.0 | 42.1 | 45.2 | 45.6 | 44.6 | 46.4 | 44.2 | 42.7 | 43.4 | 0.703 |
| **Age 65-74** | 54.7 | 50.7 | 52.0 | 53.9 | 54.0 | 54.6 | 55.8 | 53.7 | 55.2 | 55.1 | 0.003 |
| **Age 75-84** | 49.1 | 53.0 | 54.4 | 55.8 | 56.9 | 57.8 | 60.2 | 60.4 | 62.0 | 61.6 | <0.001 |
| **Age 85-99** | 43.5 | 44.6 | 47.7 | 51.0 | 51.8 | 52.8 | 53.5 | 57.7 | 59.1 | 57.8 | <0.001 |
| **Month 0-3 after discharge** |  |  |  |  |  |  |  |  |  |  |  |
| **All** | 70.8 | 70.6 | 72.3 | 74.5 | 75.4 | 76.3 | 77.7 | 78.0 | 78.9 | 79.9 | <0.001 |
| **Men** | 73.4 | 73.1 | 74.2 | 75.7 | 76.9 | 78.3 | 78.6 | 77.9 | 79.3 | 80.1 | <0.001 |
| **Women** | 67.3 | 67.6 | 70.1 | 73.0 | 73.8 | 74.1 | 76.7 | 78.1 | 78.3 | 79.7 | <0.001 |
| **Age 18-54** | 82.2 | 81.9 | 84.0 | 83.9 | 87.9 | 88.0 | 85.8 | 85.0 | 83.7 | 85.4 | 0.162 |
| **Age 55-64** | 84.3 | 80.0 | 82.3 | 84.3 | 84.9 | 84.1 | 85.5 | 87.6 | 87.5 | 87.2 | <0.001 |
| **Age 65-74** | 77.7 | 77.3 | 77.2 | 81.0 | 80.1 | 81.9 | 82.0 | 81.6 | 83.3 | 83.5 | <0.001 |
| **Age 75-84** | 69.9 | 70.7 | 72.2 | 74.3 | 75.5 | 76.9 | 78.9 | 77.7 | 79.0 | 80.7 | <0.001 |
| **Age 85-99** | 58.8 | 59.6 | 61.9 | 64.6 | 66.7 | 67.5 | 69.2 | 71.2 | 72.0 | 73.2 | <0.001 |
| **Month 3-6 after discharge** |  |  |  |  |  |  |  |  |  |  |  |
| **All** | 64.1 | 64.6 | 66.5 | 68.2 | 70.5 | 70.5 | 71.8 | 72.1 | 72.9 | 73.5 | <0.001 |
| **Men** | 66.5 | 67.3 | 67.2 | 68.1 | 72.0 | 71.3 | 71.9 | 71.7 | 72.8 | 73.0 | <0.001 |
| **Women** | 60.9 | 61.4 | 65.7 | 68.2 | 68.7 | 69.5 | 71.6 | 72.6 | 73.1 | 74.0 | <0.001 |
| **Age 18-54** | 72.9 | 71.7 | 72.8 | 73.2 | 77.1 | 78.8 | 77.3 | 78.4 | 75.0 | 76.7 | 0.004 |
| **Age 55-64** | 73.2 | 75.1 | 75.2 | 75.7 | 79.3 | 79.0 | 75.3 | 78.0 | 80.0 | 79.4 | <0.001 |
| **Age 65-74** | 69.1 | 69.9 | 72.8 | 73.4 | 74.7 | 74.2 | 75.1 | 75.5 | 76.6 | 74.5 | <0.001 |
| **Age 75-84** | 63.2 | 65.5 | 67.1 | 68.8 | 70.4 | 70.2 | 73.7 | 72.3 | 73.1 | 75.7 | <0.001 |
| **Age 85-99** | 55.9 | 53.9 | 56.1 | 59.8 | 63.5 | 64.2 | 64.7 | 66.3 | 67.5 | 67.9 | <0.001 |
| **Month 6-9 after discharge** |  |  |  |  |  |  |  |  |  |  |  |
| **All** | 63.7 | 63.6 | 65.4 | 66.9 | 68.8 | 69.4 | 70.8 | 70.6 | 72.3 | 73.1 | <0.001 |
| **Men** | 67.4 | 65.4 | 66.2 | 66.3 | 68.9 | 70.0 | 70.4 | 70.2 | 71.7 | 72.6 | <0.001 |
| **Women** | 58.9 | 61.6 | 64.5 | 67.7 | 68.8 | 68.7 | 71.2 | 71.2 | 72.9 | 73.6 | <0.001 |
| **Age 18-54** | 69.8 | 70.0 | 73.0 | 72.5 | 70.9 | 72.6 | 74.9 | 71.9 | 70.6 | 74.9 | 0.213 |
| **Age 55-64** | 74.6 | 71.1 | 73.4 | 73.0 | 77.1 | 75.1 | 75.0 | 78.7 | 77.7 | 76.8 | <0.001 |
| **Age 65-74** | 70.6 | 68.0 | 69.7 | 70.5 | 72.2 | 73.8 | 73.8 | 72.9 | 75.9 | 74.6 | <0.001 |
| **Age 75-84** | 63.2 | 64.7 | 66.1 | 67.6 | 68.9 | 70.2 | 72.3 | 70.8 | 72.7 | 75.4 | <0.001 |
| **Age 85-99** | 53.3 | 54.7 | 56.3 | 60.2 | 63.3 | 63.3 | 64.6 | 65.9 | 67.9 | 67.9 | <0.001 |
| **Month 9-12 after discharge** |  |  |  |  |  |  |  |  |  |  |  |
| **All** | 65.0 | 63.9 | 65.1 | 67.3 | 68.6 | 69.0 | 69.8 | 70.1 | 71.8 | 72.1 | <0.001 |
| **Men** | 67.5 | 65.4 | 65.6 | 67.6 | 69.0 | 68.7 | 69.1 | 70.1 | 71.5 | 71.6 | <0.001 |
| **Women** | 61.7 | 62.2 | 64.6 | 67.0 | 68.2 | 69.3 | 70.7 | 70.0 | 72.1 | 72.6 | <0.001 |
| **Age 18-54** | 69.0 | 69.5 | 69.9 | 71.2 | 73.6 | 74.1 | 69.4 | 67.1 | 72.2 | 71.0 | 0.905 |
| **Age 55-64** | 75.6 | 74.5 | 72.7 | 73.8 | 74.4 | 74.5 | 73.4 | 75.0 | 76.2 | 75.9 | 0.150 |
| **Age 65-74** | 74.0 | 68.3 | 70.3 | 73.1 | 71.0 | 73.0 | 71.7 | 73.3 | 75.4 | 73.9 | <0.001 |
| **Age 75-84** | 63.9 | 64.7 | 66.5 | 67.0 | 69.0 | 69.4 | 72.5 | 71.1 | 72.1 | 73.6 | <0.001 |
| **Age 85-99** | 54.4 | 54.5 | 55.4 | 60.4 | 63.6 | 63.3 | 64.1 | 65.6 | 67.4 | 68.2 | <0.001 |

**e-Table 7. Mineralocorticoid receptor antagonist treatment rates in patients that survived at least 12 months after discharge from a first-time hospitalization for heart failure in Sweden 2005–2014.**

| **Year** | **2005 Oct-Dec** | **2006** | **2007** | **2008** | **2009** | **2010** | **2011** | **2012** | **2013** | **2014** | **P-value for trend** |
| --- | --- | --- | --- | --- | --- | --- | --- | --- | --- | --- | --- |
| **MRA treatment rate, %** |  |  |  |  |  |  |  |  |  |  |  |
| **Month 0-3 before admission** |  |  |  |  |  |  |  |  |  |  |  |
| **All** | 9.8 | 9.7 | 9.3 | 9.7 | 9.2 | 8.9 | 9.1 | 10.2 | 10.1 | 9.6 | 0.216 |
| **Men** | 9.3 | 8.8 | 9.0 | 9.2 | 9.2 | 8.3 | 9.1 | 10.5 | 10.8 | 9.7 | <0.001 |
| **Women** | 10.4 | 10.7 | 9.8 | 10.3 | 9.1 | 9.6 | 9.1 | 9.9 | 9.4 | 9.5 | 0.038 |
| **Age 18-54** | 5.4 | 7.5 | 7.2 | 4.9 | 7.0 | 7.3 | 7.9 | 10.0 | 9.1 | 8.6 | 0.007 |
| **Age 55-64** | 12.4 | 8.0 | 11.6 | 10.0 | 10.7 | 9.4 | 11.5 | 12.2 | 11.3 | 10.4 | 0.156 |
| **Age 65-74** | 12.2 | 10.4 | 10.1 | 11.4 | 11.2 | 10.4 | 10.4 | 12.8 | 13.1 | 11.7 | 0.007 |
| **Age 75-84** | 9.7 | 9.3 | 9.0 | 10.0 | 9.4 | 8.8 | 8.9 | 9.5 | 10.6 | 10.7 | 0.036 |
| **Age 85-99** | 7.9 | 10.7 | 8.8 | 8.9 | 7.4 | 8.2 | 7.9 | 8.5 | 7.5 | 6.8 | <0.001 |
| **Month 0-3 after discharge** |  |  |  |  |  |  |  |  |  |  |  |
| **All** | 37.1 | 36.1 | 35.6 | 33.7 | 32.1 | 31.4 | 31.4 | 33.1 | 33.8 | 35.1 | <0.001 |
| **Men** | 38.5 | 36.6 | 37.0 | 35.2 | 33.6 | 32.8 | 33.1 | 35.7 | 36.1 | 37.1 | 0.407 |
| **Women** | 35.3 | 35.6 | 33.9 | 32.0 | 30.5 | 29.8 | 29.4 | 30.1 | 31.2 | 32.9 | <0.001 |
| **Age 18-54** | 40.3 | 44.5 | 43.7 | 39.5 | 39.1 | 38.1 | 41.1 | 43.1 | 45.3 | 46.7 | 0.130 |
| **Age 55-64** | 47.2 | 40.6 | 40.7 | 40.3 | 39.1 | 38.1 | 37.9 | 43.3 | 46.9 | 44.4 | 0.019 |
| **Age 65-74** | 42.5 | 37.8 | 37.7 | 38.7 | 37.0 | 36.5 | 35.8 | 40.2 | 39.8 | 42.2 | 0.013 |
| **Age 75-84** | 36.1 | 35.3 | 35.9 | 32.5 | 30.4 | 30.3 | 30.3 | 31.2 | 33.5 | 34.4 | 0.003 |
| **Age 85-99** | 29.5 | 32.6 | 29.7 | 28.1 | 27.3 | 26.1 | 25.4 | 24.8 | 23.9 | 25.7 | <0.001 |
| **Month 3-6 after discharge** |  |  |  |  |  |  |  |  |  |  |  |
| **All** | 30.1 | 29.6 | 29.2 | 27.1 | 26.3 | 25.6 | 26.4 | 27.7 | 28.9 | 29.0 | 0.157 |
| **Men** | 31.4 | 29.7 | 30.1 | 28.3 | 27.5 | 26.5 | 27.3 | 29.9 | 31.1 | 31.1 | 0.083 |
| **Women** | 28.5 | 29.5 | 28.2 | 25.7 | 25.1 | 24.5 | 25.2 | 25.2 | 26.4 | 26.5 | <0.001 |
| **Age 18-54** | 34.9 | 36.8 | 35.8 | 26.3 | 31.8 | 34.2 | 36.7 | 38.1 | 37.4 | 40.7 | 0.002 |
| **Age 55-64** | 36.8 | 31.9 | 34.5 | 32.2 | 32.6 | 32.5 | 34.8 | 36.5 | 40.4 | 38.7 | <0.001 |
| **Age 65-74** | 31.1 | 31.3 | 32.2 | 30.9 | 30.3 | 29.0 | 29.8 | 33.0 | 35.4 | 35.0 | <0.001 |
| **Age 75-84** | 29.9 | 28.9 | 28.4 | 26.5 | 24.8 | 24.0 | 24.5 | 26.2 | 27.2 | 27.3 | 0.006 |
| **Age 85-99** | 25.8 | 27.1 | 24.4 | 23.5 | 22.5 | 21.5 | 21.3 | 20.6 | 21.2 | 21.2 | <0.001 |
| **Month 6-9 after discharge** |  |  |  |  |  |  |  |  |  |  |  |
| **All** | 29.3 | 29.4 | 28.7 | 26.8 | 25.7 | 25.4 | 25.8 | 27.0 | 28.4 | 28.8 | 0.191 |
| **Men** | 30.7 | 29.6 | 29.3 | 27.7 | 27.1 | 26.9 | 27.1 | 28.7 | 30.4 | 30.8 | 0.142 |
| **Women** | 27.4 | 29.1 | 28.0 | 25.6 | 24.1 | 23.9 | 24.2 | 25.2 | 26.2 | 26.5 | <0.001 |
| **Age 18-54** | 32.6 | 34.8 | 37.0 | 31.2 | 31.4 | 32.2 | 33.8 | 38.6 | 38.6 | 41.3 | 0.001 |
| **Age 55-64** | 34.4 | 33.2 | 34.3 | 32.2 | 31.9 | 30.3 | 32.3 | 35.6 | 38.4 | 37.6 | 0.004 |
| **Age 65-74** | 33.9 | 31.0 | 30.2 | 28.3 | 28.8 | 30.2 | 30.3 | 31.7 | 35.4 | 35.3 | <0.001 |
| **Age 75-84** | 28.2 | 28.8 | 27.9 | 26.3 | 24.3 | 23.8 | 23.6 | 24.9 | 26.5 | 26.5 | <0.001 |
| **Age 85-99** | 24.5 | 26.5 | 24.4 | 23.3 | 22.1 | 21.6 | 21.5 | 20.9 | 20.8 | 21.4 | <0.001 |
| **Month 9-12 after discharge** |  |  |  |  |  |  |  |  |  |  |  |
| **All** | 29.5 | 28.9 | 28.3 | 26.6 | 25.1 | 25.0 | 25.5 | 26.9 | 27.6 | 28.5 | 0.096 |
| **Men** | 29.2 | 29.1 | 28.8 | 27.6 | 26.4 | 26.1 | 26.4 | 28.8 | 30.0 | 30.5 | 0.035 |
| **Women** | 29.9 | 28.7 | 27.8 | 25.4 | 23.6 | 23.9 | 24.5 | 24.7 | 24.9 | 26.1 | <0.001 |
| **Age 18-54** | 26.4 | 33.2 | 32.8 | 28.6 | 30.9 | 30.7 | 33.3 | 36.5 | 33.0 | 39.9 | <0.001 |
| **Age 55-64** | 35.5 | 34.4 | 33.0 | 31.7 | 29.8 | 31.7 | 32.1 | 37.0 | 38.9 | 38.3 | <0.001 |
| **Age 65-74** | 33.7 | 30.1 | 31.1 | 29.7 | 27.7 | 28.1 | 28.6 | 32.4 | 34.3 | 34.5 | <0.001 |
| **Age 75-84** | 29.4 | 27.9 | 27.8 | 25.9 | 23.8 | 23.7 | 23.9 | 24.1 | 26.1 | 26.8 | 0.002 |
| **Age 85-99** | 24.7 | 26.4 | 24.1 | 23.0 | 22.3 | 21.6 | 21.6 | 20.7 | 20.2 | 20.7 | <0.001 |

MRA, mineralocorticoid receptor antagonist.

**e-Table 8. Digitalis treatment rates in patients that survived at least 12 months after discharge from a first-time hospitalization for heart failure in Sweden 2005–2014.**

| **Year** | **2005 Oct-Dec** | **2006** | **2007** | **2008** | **2009** | **2010** | **2011** | **2012** | **2013** | **2014** | **P-value for trend** |
| --- | --- | --- | --- | --- | --- | --- | --- | --- | --- | --- | --- |
| **Digitalis treatment rate, %** |  |  |  |  |  |  |  |  |  |  |  |
| **Month 0-3 before admission** |  |  |  |  |  |  |  |  |  |  |  |
| **All** | 12.7 | 12.6 | 10.7 | 9.3 | 9.3 | 9.0 | 8.2 | 8.1 | 7.3 | 6.6 | <0.001 |
| **Men** | 10.8 | 11.5 | 9.5 | 7.9 | 7.7 | 7.7 | 6.8 | 6.8 | 5.9 | 5.0 | <0.001 |
| **Women** | 15.2 | 14.0 | 12.2 | 10.9 | 11.2 | 10.5 | 9.9 | 9.5 | 8.9 | 8.5 | <0.001 |
| **Age 18-54** | 3.9 | 4.1 | 3.2 | 3.2 | 2.3 | 2.8 | 3.7 | 2.2 | 2.6 | 1.6 | 0.016 |
| **Age 55-64** | 7.0 | 6.2 | 6.2 | 5.2 | 5.5 | 4.7 | 5.6 | 4.3 | 2.8 | 4.0 | <0.001 |
| **Age 65-74** | 11.4 | 11.5 | 10.2 | 8.1 | 7.7 | 8.3 | 7.0 | 7.0 | 7.7 | 5.3 | <0.001 |
| **Age 75-84** | 14.2 | 13.6 | 11.8 | 10.2 | 10.8 | 10.3 | 9.1 | 9.8 | 8.4 | 7.2 | <0.001 |
| **Age 85-99** | 15.9 | 16.4 | 13.3 | 11.7 | 11.3 | 10.5 | 9.8 | 9.3 | 8.2 | 8.7 | <0.001 |
| **Month 0-3 after discharge** |  |  |  |  |  |  |  |  |  |  |  |
| **All** | 22.3 | 20.9 | 18.8 | 16.2 | 15.9 | 15.8 | 15.1 | 14.2 | 13.9 | 12.3 | <0.001 |
| **Men** | 20.5 | 19.5 | 17.6 | 15.0 | 14.2 | 14.5 | 13.8 | 13.0 | 12.9 | 11.2 | <0.001 |
| **Women** | 24.6 | 22.6 | 20.2 | 17.7 | 17.8 | 17.3 | 16.6 | 15.6 | 15.1 | 13.4 | <0.001 |
| **Age 18-54** | 15.5 | 16.2 | 12.8 | 13.2 | 14.6 | 13.9 | 12.7 | 8.8 | 7.9 | 8.4 | <0.001 |
| **Age 55-64** | 23.7 | 18.9 | 19.0 | 13.5 | 14.6 | 14.4 | 15.4 | 14.1 | 14.0 | 12.9 | <0.001 |
| **Age 65-74** | 20.6 | 20.1 | 18.4 | 16.1 | 15.5 | 15.9 | 14.5 | 14.0 | 15.0 | 12.5 | <0.001 |
| **Age 75-84** | 24.2 | 20.5 | 19.7 | 16.6 | 16.3 | 15.8 | 16.1 | 15.9 | 15.0 | 12.9 | <0.001 |
| **Age 85-99** | 21.4 | 23.8 | 19.3 | 17.6 | 16.3 | 16.5 | 14.7 | 13.6 | 12.9 | 11.9 | <0.001 |
| **Month 3-6 after discharge** |  |  |  |  |  |  |  |  |  |  |  |
| **All** | 19.1 | 17.7 | 15.4 | 14.3 | 13.5 | 13.6 | 12.9 | 11.9 | 11.5 | 10.4 | <0.001 |
| **Men** | 17.3 | 16.3 | 14.1 | 12.8 | 11.7 | 12.3 | 11.6 | 10.4 | 10.5 | 9.1 | <0.001 |
| **Women** | 21.5 | 19.4 | 16.8 | 15.9 | 15.6 | 15.0 | 14.4 | 13.7 | 12.8 | 11.8 | <0.001 |
| **Age 18-54** | 12.4 | 11.8 | 10.1 | 11.1 | 10.4 | 9.2 | 8.2 | 5.5 | 5.9 | 5.5 | <0.001 |
| **Age 55-64** | 16.1 | 13.8 | 14.0 | 11.5 | 10.5 | 11.8 | 12.8 | 10.5 | 10.1 | 9.9 | <0.001 |
| **Age 65-74** | 19.3 | 16.8 | 14.2 | 13.3 | 12.6 | 12.9 | 11.4 | 11.8 | 12.3 | 10.1 | <0.001 |
| **Age 75-84** | 19.8 | 18.0 | 16.5 | 14.8 | 14.5 | 14.1 | 13.9 | 13.9 | 12.6 | 11.2 | <0.001 |
| **Age 85-99** | 20.8 | 20.8 | 16.4 | 15.9 | 14.6 | 15.0 | 13.5 | 11.6 | 11.3 | 10.7 | <0.001 |
| **Month 6-9 after discharge** |  |  |  |  |  |  |  |  |  |  |  |
| **All** | 18.7 | 17.5 | 15.6 | 13.8 | 13.3 | 13.0 | 12.3 | 11.6 | 11.1 | 10.3 | <0.001 |
| **Men** | 16.4 | 16.4 | 14.4 | 12.6 | 11.7 | 11.4 | 10.8 | 10.5 | 10.0 | 9.0 | <0.001 |
| **Women** | 21.8 | 18.8 | 17.0 | 15.2 | 15.2 | 14.7 | 14.0 | 13.0 | 12.4 | 11.8 | <0.001 |
| **Age 18-54** | 10.9 | 11.6 | 9.3 | 9.8 | 8.9 | 8.5 | 6.9 | 5.5 | 5.4 | 5.8 | <0.001 |
| **Age 55-64** | 16.1 | 13.5 | 14.6 | 11.5 | 10.5 | 10.2 | 11.6 | 10.5 | 8.8 | 8.6 | <0.001 |
| **Age 65-74** | 17.2 | 16.6 | 15.3 | 12.6 | 12.7 | 13.0 | 11.7 | 11.9 | 12.8 | 10.2 | <0.001 |
| **Age 75-84** | 20.3 | 17.9 | 16.4 | 13.9 | 14.2 | 13.3 | 13.4 | 13.6 | 11.7 | 11.0 | <0.001 |
| **Age 85-99** | 20.3 | 20.3 | 16.5 | 16.1 | 14.5 | 14.2 | 12.7 | 10.8 | 11.2 | 11.0 | <0.001 |
| **Month 9-12 after discharge** |  |  |  |  |  |  |  |  |  |  |  |
| **All** | 18.7 | 17.1 | 15.3 | 14.1 | 12.9 | 13.1 | 12.4 | 11.3 | 11.3 | 10.2 | <0.001 |
| **Men** | 16.2 | 15.5 | 14.4 | 12.9 | 11.1 | 11.4 | 11.2 | 9.7 | 9.9 | 9.0 | <0.001 |
| **Women** | 21.9 | 19.0 | 16.2 | 15.6 | 14.8 | 15.0 | 13.9 | 13.2 | 12.8 | 11.5 | <0.001 |
| **Age 18-54** | 12.4 | 11.1 | 9.4 | 8.2 | 7.5 | 8.8 | 6.8 | 4.9 | 5.2 | 5.0 | <0.001 |
| **Age 55-64** | 14.7 | 13.7 | 13.5 | 11.7 | 10.3 | 8.9 | 11.8 | 10.0 | 8.6 | 8.6 | <0.001 |
| **Age 65-74** | 17.6 | 15.9 | 15.8 | 13.8 | 12.2 | 13.3 | 10.9 | 11.0 | 12.2 | 10.6 | <0.001 |
| **Age 75-84** | 20.2 | 17.3 | 16.0 | 14.3 | 14.2 | 13.6 | 13.5 | 13.4 | 12.0 | 10.7 | <0.001 |
| **Age 85-99** | 20.3 | 20.3 | 16.0 | 16.2 | 13.6 | 14.6 | 13.6 | 11.0 | 11.7 | 10.8 | <0.001 |
